# Anamnese und klinische Untersuchung in der Notfall- und Intensivmedizin

**DOI:** 10.1007/s00063-020-00731-x

**Published:** 2020-09-03

**Authors:** C. Steinkellner, C. Schlömmer, M. Dünser

**Affiliations:** Klinik für Anästhesiologie und Intensivmedizin, Kepler Universitätsklinikum, Medizinische Fakultät, Johannes Kepler Universität, Krankenhausstraße 9, 4020 Linz, Österreich

**Keywords:** Kritische Erkrankung, Diagnostische Techniken und Prozeduren, Notfallmedizin, Notaufnahme, Diagnose, Critical illness, Diagnostic techniques and procedures, Emergency medicine, Emergency department, Diagnosis

## Abstract

Die Anamnese und die klinische Untersuchung stellen das traditionelle Grundwerkzeug der ärztlichen Tätigkeit bei der Diagnosefindung dar. Sowohl Anamnese als auch klinische Untersuchung haben in der modernen Notfall- und Intensivmedizin zu Unrecht an praktischem Stellenwert verloren. Bei der Diagnosefindung des akut oder kritisch kranken Patienten müssen die Reihenfolge, Technik und Fokussierung der Anamnese und klinischen Untersuchung an die individuelle Situation und den Zustand des Patienten angepasst werden. In diesem Artikel wird die nach wie vor zentrale Bedeutung der Anamnese und klinischen Untersuchung bei der Diagnosefindung des Notfall- und Intensivpatienten praktisch evaluiert und beleuchtet.

**Zusatzmaterial online:**

Die Onlineversion dieses Beitrags (10.1007/s00063-020-00731-x) enthält ein leitsymptombasiertes, strukturiertes Anamneseschema am Beispiel der „respiratorischen Insuffizienz“. Beitrag und Zusatzmaterial stehen Ihnen auf www.springermedizin.de zur Verfügung. Bitte geben Sie dort den Beitragstitel in die Suche ein, das Zusatzmaterial finden Sie beim Beitrag unter „Ergänzende Inhalte“.

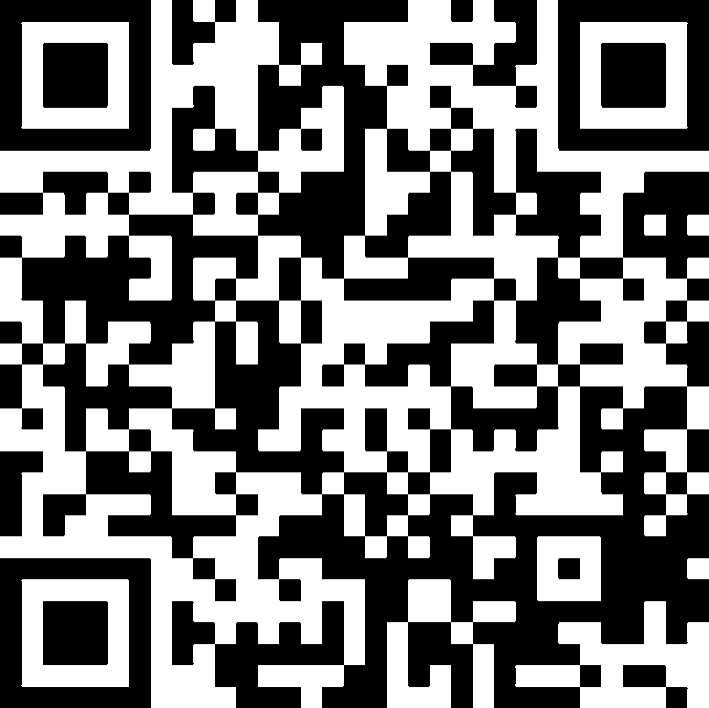

## Abstract (en)

Medical history taking and the clinical examination are the traditional medical tools in making a diagnosis. Both the medical history taking and clinical examination have wrongly lost their practical value in modern emergency and intensive care medicine. When diagnosing an acutely or critically ill patient, the sequence, technique and focus of history taking and the clinical examination must be adapted to the individual situation and the condition of the patient. In this article the continuing central importance of both techniques in the diagnostics of emergency and intensive care patients is practically evaluated and discussed.

## Hintergrund

Die Anamnese und klinische Untersuchung stellen das traditionelle Grundwerkzeug der ärztlichen Tätigkeit bei der Diagnosefindung dar, auch in der Notfall- und Intensivmedizin. Eine im Jahr 1975 publizierte, englische Observationsstudie von Hampton et al. zeigte, dass bei 82,5 % der Patienten, die in einer allgemeinen, internistischen Ambulanz gesehen wurden, die korrekte Diagnose allein durch das Anamnesegespräch gestellt werden konnte. Der Beitrag der klinischen Untersuchung sowie der laborchemischen oder bildgebenden Zusatzuntersuchungen zur Diagnosesicherung wurden jeweils mit 8,8 % angegeben [1]. Diese Ergebnisse konnten mit nahezu identischen Zahlen in den Jahren 1992 und 2000 durch andere Autoren reproduziert werden [2, 3]. Somit scheinen diese Eckdaten auch heute noch für die Mehrzahl der stabilen, wachen und kommunikationsfähigen Patienten zuzutreffen. Die Anamnese und klinische Untersuchung bilden daher die Grundlagen für die medizinische Diagnosestellung und stehen im Ablaufdiagramm zur Diagnosefindung an oberster Stelle.

Die Notfall- und Intensivmedizin stellt besondere Anforderungen an den Arzt und dessen diagnostische Fähigkeiten. Nicht oder nur eingeschränkt kommunikationsfähige Patienten und der Zeitdruck, eine vitale Bedrohung abzuwenden, erschweren die Übertragbarkeit der Ergebnisse zur Anamnese und klinischen Untersuchung auf die Versorgung von Notfall- und Intensivpatienten. Zusätzlich liefern medizintechnische Geräte (z. B. Vitaldatenmonitore, Beatmungsgeräte) rasch und kontinuierlich Informationen über den Zustand des Patienten. In diesem Spannungsfeld verloren die Anamnese und klinische Untersuchung in der Notfall- und Intensivmedizin zusehends an praktischem Stellenwert.

Anamnese und klinische Untersuchung haben unbegründeterweise an praktischem Stellenwert verloren

So fand eine Befragungsstudie aus den USA, dass Intensivmediziner (Fach‑/Oberärzte) ihre Patienten nur in etwa 10 % der Fälle auch klinisch untersuchten [4]. Welche Bedeutung kommt der Anamnese und klinischen Untersuchung in der Notfall- und Intensivmedizin überhaupt noch zu? Im nachfolgenden Manuskript soll die nach wie vor zentrale Bedeutung der Anamnese und klinischen Untersuchung bei der Diagnosefindung des Notfall- und Intensivpatienten praktisch evaluiert und beleuchtet werden.

## Prioritäten setzen oder: „Treat first what kills first!“

**Figure 1:**
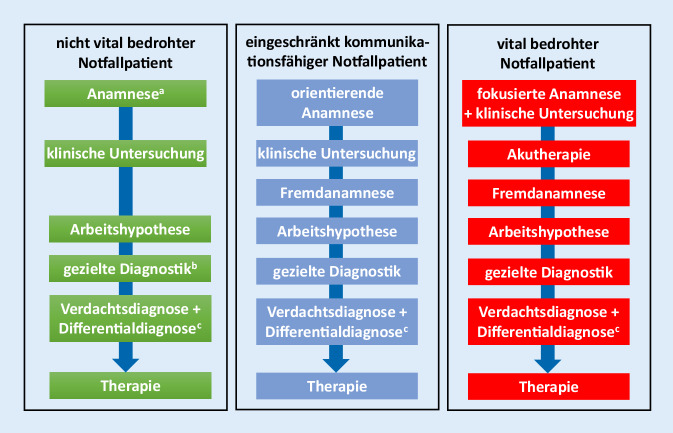

Grundsätzlich unterscheiden sich akut oder kritisch erkrankte Patienten nicht von jenen Patienten, die – wie bereits beschrieben – in die viel zitierte Studie von Hampton et al. eingeschlossen wurden. Sie alle leiden an einer Erkrankung, die jedoch im Gegensatz zu nichtkritisch kranken Patienten akut zu einer potenziellen oder tatsächlichen Bedrohung der Vital- und/oder Organfunktionen führte. Dieser Umstand bedeutet, dass die Reihung der diagnostischen und therapeutischen Maßnahmen priorisiert und entsprechend geändert werden muss, nicht aber fallengelassen werden darf. Die klassische diagnostische und therapeutische Vorgehensweise sowie eine mögliche Adaptierung des Vorgehens bei Notfallpatienten mit eingeschränkter Kommunikationsmöglichkeit bzw. solchen mit akut lebensbedrohlichen Zuständen ist in Abb. 1 gezeigt.

Wichtig ist in allen Fällen, dass die einzelnen Schritte der klassischen Vorgehensweise (z. B. Fremdanamnese bei einem bewusstseinsgestörten Patienten) nicht verworfen oder durch ein liberales Screening (z. B. ungerichtetes Laborpanel) bzw. eine unspezifische Therapie (z. B. liberale Antibiotikatherapie trotz fehlendem Infektionshinweis) ersetzt, sondern zu einem späteren Zeitpunkt nachgeholt werden. Neben dem Verlust der (differenzial)diagnostischen Fähigkeiten birgt eine ungerichtete „Screening“-Diagnostik das Risiko, einerseits individuell erforderliche Untersuchungen gar nicht durchzuführen und andererseits Untersuchungsergebnisse ohne pathologische Bedeutung zu identifizieren. Entsprechend häufig sind mit einem solchen undifferenzierten Vorgehen Fehldiagnosen und unnötig hohe Kosten assoziiert. Eine ungerichtete Diagnostik impliziert in vielen Fällen auch eine ungerichtete Therapie, was wiederum das Risiko birgt, die eigentliche Grundpathologie nicht zu behandeln und andererseits Nebenwirkungen infolge einer Übertherapie zu verursachen.

## Die Anamnese

*„Always listen to the patient, he might be telling you the diagnosis.“* Sir William Osler

Bei Patienten mit Intoxikationen ist die Eigenanamnese nicht sicher verwertbar

Die Anamnese ist bei nichttraumatologischen Notfallpatienten der wichtigste Schritt in der Diagnosefindung und wird in Eigen- und Fremdanamnese differenziert. Während bei der Eigenanamnese der Patient selbst die Krankheitsgeschichte wiedergeben kann, wird bei der Fremdanamnese eine dritte Person (z. B. Angehöriger) gefragt, über die Erkrankung/den Erkrankungsverlauf des Patienten Auskunft zu geben. Weiterhin können Personen, die den Patienten medizinisch betreuen (z. B. Pflegeperson, Betreuer, Arzt) oder ein Notfallereignis unmittelbar beobachtet haben (z. B. Augenzeugen), wichtige Informationen zum Erkrankungs- oder Unfallhergang liefern. Bei Notfallpatienten, deren Kommunikationsfähigkeit eingeschränkt ist, kann entweder nur eine orientierende Anamnese (z. B. bei Patienten mit Atemnot) oder gar keine Eigenanamnese (z. B. bewusstseinsgestörte Patienten) erhoben werden. Bei Patienten mit Intoxikationen ist die Eigenanamnese typischerweise nicht sicher verwertbar. So zeigte eine finnische Untersuchung an 51 Patienten mit Medikamenten- bzw. Substanzintoxikation, dass nur bei 27 % die eigenanamnestischen Angaben zur Art und Menge der eingenommenen Substanzen auch tatsächlich mit den Laboranalysen übereinstimmten [5]. Entsprechende Vorsicht ist daher bei der Interpretation der Eigenanamnese von Patienten nach Medikamenteneinnahmen in suizidaler Absicht walten zu lassen.

Die Eigenanamnese sollte auch in der Akut- und Notfallmedizin zu Beginn primär frei geführt werden. Dies bedeutet, dass der Patient nach einer offenen Frage („Was führt Sie zu uns?“ oder „Wie können wir Ihnen helfen?“) berichten kann, warum er/sie medizinische Hilfe sucht. Diese freie Anamnese zielt darauf ab, das Leitsymptom (z. B. Luftnot, neu aufgetretenes neurologisches Defizit, Schmerzen) zu identifizieren. Die Angst vieler Notfall- und Intensivmediziner, durch offene Fragen in unendliche Geschichten und Details verwickelt zu werden, scheint unbegründet zu sein. So zeigte eine Analyse, dass die meisten Patienten – wenn sie nicht unterbrochen werden – auf eine offene Frage nach ihrem Leitsymptom, dieses innerhalb von weniger als 60 s beschreiben [6].

Die korrekte und strukturierte Anamneseerhebung erfordert ein breites medizinisches Fachwissen

Nach Identifikation des Leitsymptoms empfiehlt es sich, die Anamnesetechnik zu wechseln und gezielt Qualitäten und Risikofaktoren des beschriebenen Leitsymptoms abzufragen. Die korrekte und strukturierte Anamneseerhebung setzt viel Erfahrung voraus und erfordert ein breites medizinisches Fachwissen. Die Fähigkeit, abhängig vom Leitsymptom die richtigen Fragen auch in einer Stresssituation zu stellen, wird dem Notfallmediziner nicht in die Wiege gelegt, sondern muss (wie alles andere in der Medizin) erlernt werden. Hierzu empfiehlt es sich für häufige Leitsymptome, entsprechende Checklisten bereitzuhalten (Tab. 1). Jede Anamnese sollte außerdem, zumeist nach Abschluss des freien und fokussierten Teils, einen allgemeinen Teil enthalten, der bei jedem Patienten abgefragt wird (Tab. 2). Bei Notfallpatienten muss dieser Teil häufig zu einem späteren Zeitpunkt nachgeholt werden. Die Frage nach einer bekannten Allergie sollte jedoch selbst bei zeitkritischen Patienten immer mit der ersten Anamneseerhebung gestellt werden.

**Table 1:** 

**Herz-Kreislauf-Stillstand**		
**Anamnese möglichst früh erheben (z.** **B. bereits von Angehörigen am Weg zum Patienten)! Augenzeugen, Familienangehörige, Pflegepersonen befragen!**		
**Ja**	**Nein**	**Patientenzustand und -wünsche**
□	□	Ist der Patientenwunsch bezüglich Reanimationsmaßnahmen bekannt? *wenn ja*, entsprechend vorgehen
□	□	Alter des Patienten?
□	□	Bekannte schwere Vorerkrankung (z. B. metastasiertes Malignom, fortgeschrittene Herzinsuffizienz, schwere chronisch-obstruktive Lungenerkrankung (COPD), fortgeschrittene Demenz)? *wenn ja*, schlechtes prognostisches Zeichen
**Ja**	**Nein**	**Dauer No-flow- und Low-flow-Zeit**
□	□	Wurde der Kollaps beobachtet? *wenn nein*, schlechtes prognostisches Zeichen *wenn ja*, genauen Zeitpunkt des Kollapses erfragen
□	□	Laienreanimation erfolgt? *wenn nein*, schlechtes prognostisches Zeichen
□	□	Zeit zwischen Kollaps und Beginn der Laienreanimation? *wenn >10* *min*, schlechtes prognostisches Zeichen
□	□	Dauer Laienreanimation?
**Ja**	**Nein**	**Prodromi unmittelbar vor Kollaps**
□	□	Kopfschmerzen? *wenn ja*, erwäge Subarachnoidalblutung
□	□	Thoraxschmerz? *wenn ja*, erwäge Myokardinfarkt, Lungenembolie, Aortendissektion
□	□	Dyspnoe? *wenn ja*, erwäge Myokardinfarkt, Lungenembolie, Hypoxie, Hyperkapnie

**Table 2:** 

Dauermedikation^a^
Allergien
Vorbefunde aus früheren Krankenhausaufenthalten oder Arztbesuchen
Risikofaktoren (relevant für Leitsymptom): z. B. Nikotin, Alkohol, Drogen, Familienanamnese, Hypertonie, Hyperlipidämie, Diabetes mellitus
Reise- und Auslandsanamnese
Vegetative Anamnese (relevant für Leitsymptom): z. B. B‑Symptomatik, Fieber, Miktionsbeschwerden, Defäkationsbeschwerden
Sozial- (Berufs-) und Sexualanamnese
Familienanamnese (Todesursachen und/oder schwere Erkrankungen bei Vater, Mutter oder Geschwistern)
Körperlicher Zustand vor dem Akutereignis
Patientenverfügung und Therapiewünsche
Kontakt zu Angehörigen bzw. Ansprechpartnern

^a^Die Dauermedikation ist ein wichtiger Anamnesebestandteil und muss im Regelfall schriftlichen Unterlagen entnommen werden (da gerade ältere Patienten bei Einnahme mehrere Medikamente diese nicht mit korrektem Namen und in der korrekten Dosierung wiedergeben können. „Sie wissen schon, Herr Doktor, die gelben Tabletten!“). Die Liste der regelmäßig verordneten bzw. eingenommenen Medikamente kann wichtige Aufschlüsse über Krankheiten geben, die vom Patienten bei der Frage nach Vorerkrankungen nicht erwähnt werden. Fragen Sie nicht nur nach der verordneten Dauermedikation, sondern auch, ob diese Medikamente auch regelmäßig eingenommen werden!

Die Erhebung der (Fremd‑)Anamnese stellt eine der wichtigsten Aufgaben des prähospital tätigen Arztes dar. Während viele Tätigkeiten eines Notarztes an entsprechend ausgebildetes Rettungsdienstpersonal delegiert werden können, ist die Anamneseerhebung eine ärztliche Aufgabe. In vielen Fällen (z. B. bei Notfällen in öffentlichen Räumen) stellt die prähospitale Behandlungsphase die einzige Möglichkeit dar, eine Fremdanamnese einzuholen. Bei der Versorgung von kritisch kranken Patienten kann dies jedoch manchmal aus Zeitdruck nicht möglich sein. In solchen Situationen hat es sich bewährt, die Telefonnummer von Angehörigen, Pflegepersonen oder Augenzeugen zu dokumentieren, um diese dem Behandlungsteam im Krankenhaus zu übergeben, damit die Fremdanamnese im Intervall eingeholt werden kann.

Die Anamneseerhebung ist eine ärztliche Aufgabe

Gerade bei der notfallmedizinischen Versorgung von nichtentscheidungsfähigen, geriatrischen Patienten nimmt die Fremdanamnese einen essenziellen Stellenwert in der Diagnosefindung und weiteren Behandlungsplanung ein. Bei diesen Patienten ist es besonders wichtig, Informationen von Angehörigen, Pflegepersonen oder Betreuern über deren funktionellen Zustand vor der akuten Erkrankung sowie allfällig geäußerte Therapiewünsche (insbesondere intensivmedizinische Maßnahmen betreffend) einzuholen. Auch wenn solche Gespräche im Idealfall unter geordneten Bedingungen und persönlich geführt werden sollten, müssen diese gerade von der Notaufnahme aus immer wieder telefonisch abgewickelt werden. Eine Verzögerung bei der Erhebung dieser Informationen kann zu therapeutischen Maßnahmen führen, die weder vom Patienten gewünscht noch aus medizinischen und ethischen Gesichtspunkten gerechtfertigt sind.

Eine weitere Besonderheit bei der Anamneseerhebung in der Notfallmedizin stellt die Tatsache dar, dass viele Patienten, aber auch Angehörige und Dritte in der Akutsituation unvollständige oder missverständliche Angaben machen. Jeder Notfallmediziner kennt die Situation, dass ein kurze Zeit später geführtes Anamnesegespräch verglichen mit dem zuvor selbst geführten Anamnesegespräch neue bzw. klärende Gesichtspunkte ans Tageslicht bringt. Die Instabilität der Anamnese ist daher gerade in der Notfallmedizin ein häufig anzutreffendes Phänomen [7]. Um diese potenzielle Fallgrube zu umgehen, empfiehlt es sich, sofern möglich die Anamnese über das Akutereignis im Intervall (z. B. nach einigen Stunden) zu wiederholen.

Die Instabilität der Anamnese ist ein häufig anzutreffendes Phänomen

Selbst eine detaillierte und umfassende Anamnese verliert ihren Wert für die weitere Behandlung (gerade auf der Intensivstation), wenn diese bzw. relevante Teile davon nicht weitergegeben werden. Die Dokumentation relevanter Inhalte des Anamnesegesprächs (wichtig: dies inkludiert auch gezielte Fragen, die mit „nein“ beantwortet wurden) in der Patientenakte sind daher von ebenso großer Bedeutung wie die Anamneseerhebung selbst.

## Die klinische Untersuchung

*„For most diagnoses all that is needed is an ounce of knowledge, an ounce of intelligence and a pound of thoroughness.“ *Anonymes arabisches Zitat

**Figure 2:**
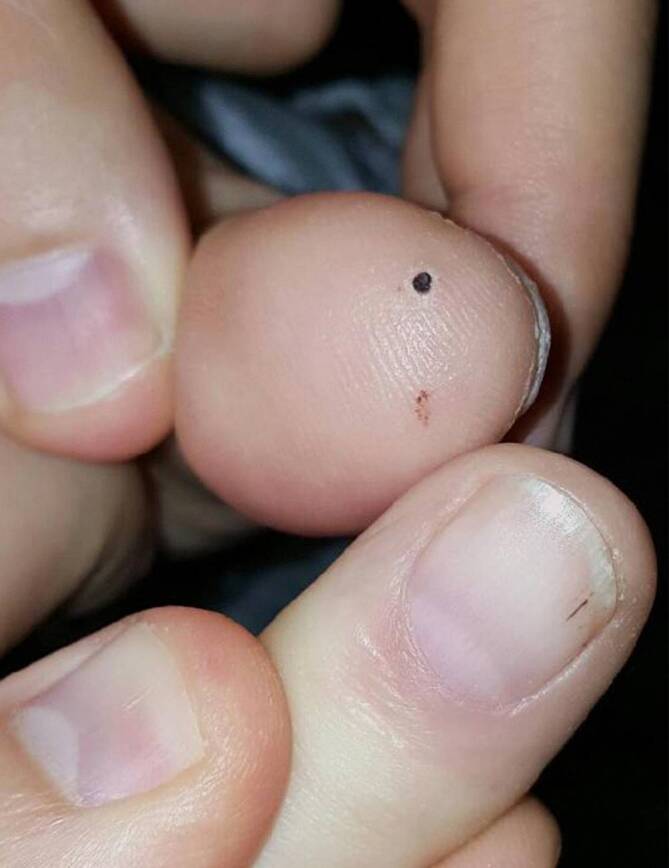

Bei der klinischen Untersuchung in der Notfall- und Intensivmedizin gilt es, einige grundsätzliche Prinzipien einzuhalten. Das erste und wohl wichtigste Prinzip ist Genauigkeit (Abb. 2). Gerade in einer Notfallsituation bedeutet Genauigkeit nicht, alles und jedes zu untersuchen, sondern eben diese Untersuchungsschritte fokussiert durchzuführen, die entsprechend des Beschwerdebilds des Patienten am wahrscheinlichsten die wichtigsten Informationen liefern werden. So einleuchtend diese Vorgabe klingt, so schwierig ist es, dies auch in einer Stresssituation umzusetzen.

Das erste und wichtigste Prinzip der klinischen Untersuchung ist die Genauigkeit

**Figure 3:**
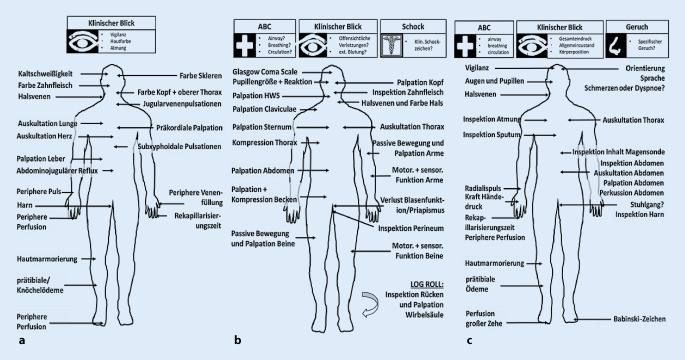

Aus diesem Grund haben sich leitsymptombasierte, strukturierte Untersuchungsschemen bewährt (Abb. 3). Solche Schemata müssen in der täglichen Routine eingeübt werden, um dann in der Notfallsituation auch prompt „abrufbar“ bzw. anwendbar zu sein. Gerade in der Notfallmedizin ist es außerdem wichtig, trotz zahlreicher externer und interner Stressoren ausreichend Aufmerksamkeit auf die Interpretation der einzelnen Untersuchungsschritte zu legen. Persönliche Erfahrungen zeigen, dass gerade in kritischen Situationen pathologische Untersuchungsergebnisse trotz einer adäquaten und strukturierten Untersuchungstechnik übersehen bzw. ignoriert werden.

Die klinische Untersuchung in der Intensivmedizin wird im Regelfall unter weit geordneteren und ruhigeren Bedingungen durchgeführt als jene in der prähospitalen Notfallmedizin oder in der Notaufnahme. Diese Umstände müssen bewusst genutzt werden, um allfällige klinische Zeichen, die bislang nicht erkannt wurden, zu identifizieren. Auch wenn die tägliche klinische Untersuchung des kritisch kranken Patienten auf der Intensivstation verglichen mit der Erstuntersuchung typischerweise nur wenige neue Untersuchungsbefunde liefert, darf ihr Wert nicht unterschätzt werden oder die Untersuchung gar ganz fallengelassen werden. In Tab. 3 sind häufige Fehler bei der Durchführung der klinischen Untersuchung (gerade auf der Intensivstation) zusammengefasst.

**Table 3:** 

Keine vollständige Entkleidung des Patienten zur Untersuchung (unter Einhaltung der Privatsphäre)
Keine Inspektion von Wunden und Einstichstellen invasiver Katheter, weil die Verbände oder Abdeckungen neu angelegt wurden
Keine Entfernung der Pulsoxymetrie, um alle 10 Finger nach klinischen Zeichen einer Endokarditis oder nach anderen klinisch-pathologischen Befunden zu inspizieren
Keine Inspektion und Begutachtung von Körper- oder Drainageflüssigkeiten
Keine systematische klinisch-körperliche Untersuchung aufgrund verschiedener Umstände (z. B. Zeitdruck, Arbeitspensum, Warteliste in der Ambulanz)
Keine erneute Untersuchung des Patienten, da keine neuen Befunde oder Befundveränderungen erwartet werden
Keine Inspektion von Auflageflächen (z. B. Occiput, Fersen, Sakralregion, Glutealregion)
Keine Durchführung gewisser Untersuchungen trotz bestehender Indikation (z. B. rektale oder vaginale Untersuchung)

### Stellenwert des ersten Eindrucks

*„Don’t touch the patient – state first what you see, cultivate your powers of observation.“* Sir William Osler

Der erste Blick auf einen Patienten bzw. der erste Eindruck von diesem liefert in der Notfallmedizin wichtige Informationen und ist von essenzieller Bedeutung. In der prähospitalen Notfallmedizin beginnt dieser erste Eindruck bereits vor dem ersten Blick auf den Patienten. So können z. B. bereits beim Betreten der Wohnung die 3 Zeitungen auf der Fußmatte einen Hinweis darauf geben, dass der Patient seit einigen Tagen die Wohnung nicht mehr verlassen hat. Ein aufmerksamer Blick sowie Geruchssinn nach Betreten der Wohnräumlichkeiten sagt viel über die sozialen Umstände des Patienten aus. Ähnlich wichtige Informationen liefert z. B. der Weg zum Unfallort, der nicht nur für die eigene Sicherheit von vitaler Bedeutung sein kann, sondern bereits viel über das zu erwartende Verletzungsmuster bzw. die Verletzungsschwere des Patienten aussagt. Ein häufiger Fehler ist es, dem allgemeinen gesellschaftlichen Druck, dass Notfallteams stets zum Einsatzort laufen müssen, nachzukommen und dabei wichtige Eindrücke und Informationen rund um den Einsatzort zu übersehen.

Der erste Eindruck des Patienten – sowohl prähospital, in der Notaufnahme oder bei Aufnahme auf der Intensivstation – umfasst nicht nur visuelle, sondern auch akustische und olfaktorische Sinneswahrnehmungen (Tab. 4). Wichtig ist dabei zu beachten, dass gewisse Patientengruppen notorisch schwer zu beurteilen sind und Fehleinschätzungen beim ersten Eindruck häufig sein können. Hier sind insbesondere geriatrische Patienten (mit neurologischen Problemen), hämatoonkologische Patienten sowie Kinder zu erwähnen. Im Gegensatz zu den vorher genannten Personengruppen besteht dabei das Risiko, dass der Zustand des Kinds besser eingeschätzt wird als dieser wirklich ist.

**Table 4:** 

Visuelle Eindrücke	Akustische Eindrücke	Olfaktorische Eindrücke
Allgemeinzustand (z. B. jung/gesund, kritisch krank, chronisch krank, „frail“^a^, moribund)	Atemgeräusche (z. B. Gurgeln, Röcheln, feuchte Rasselgeräusche, spastisches Exspirium)	„Meläna“-Geruch (z. B. gastrointestinale Blutung)
Gesichtsfarbe (z. B. rot, blass, zyanotisch, grau)	Sprache (z. B. Sprechdyspnoe bzw. Fähigkeit, in ganzen Sätzen zu sprechen)	Leber/faule Eier (z. B. hepatische Enzephalopathie, chron. Leberversagen)
Stirnschweiß (ja/nein)	Verbale Äußerungen (z. B. Schmerzen, Agitation, Psychose)	Azeton (z. B. Urämie)
Gesichtsausdruck und Blick (z. B. Angst, Schmerz, Atemnot, Vigilanzstörung)		Harnwegsinfektion
Gehfähigkeit (geht/steht alleine, geht/steht mit Unterstützung, sitzt/liegt^b^)		Typische Gerüche bei Intoxikationen (z. B. Foetor aethylicus)
Körperposition (z. B. Hochlagerung Oberkörper, Arme abgestützt, schlaffer Tonus, Deformationen, Hand auf der Brust^c^, neuropathologische Körperpositionen)		
Bewegungen (z. B. Agitation, epileptischer Anfall, neuropathologische Bewegungen wie Dezerebrierung)		
Atemmuster und -arbeit (physiologisch, paradox bei verlegtem Atemweg, obstruktiv, restriktiv)		
Offensichtliche Verletzungen		
Typische Erkrankungszeichen (z. B. Fazialisparese, Leberstigmata, rheumatologische Erkrankungen)		

^a^Siehe auch Clinical Frailty Scale [10]

^b^Eine afrikanische Studie zeigte, dass der erste Eindruck über die Gehfähigkeit bei Präsentation in der Notaufnahme wertvolle prognostische Informationen liefern kann [11]

^c^Sog. Levine-Zeichen als möglicher Hinweis auf ein akutes Koronarsyndrom

Der erste Eindruck umfasst auch akustische und olfaktorische Sinneswahrnehmungen

**Figure 4:**
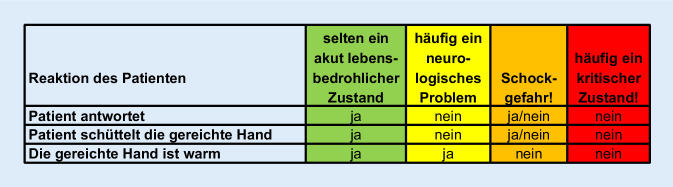

Selbst in Notfallsituationen dürfen Empathie, Höflichkeit und Menschlichkeit in der Medizin nicht verloren gehen. Ein wichtiges Symbol dessen ist, dass das Behandlungsteam den Patienten begrüßt, sich vorstellt und der Teamleiter dieser/m die Hand reicht. Die Reaktion des Patienten auf diese Gesten liefert weitere wichtige Informationen, die unserer Erfahrung nach auch eine gewisse Kategorisierung des Notfallpatienten erlauben (Abb. 4).

Nach der Verarbeitung und Interpretation des ersten Eindrucks gilt es, den Patienten auch während der Anamneseerhebung klinisch zu beobachten. Dabei können Aspekte wie die Sprache des Patienten (z. B. Dysarthrie? Aphasie? Sprechdyspnoe?) oder dessen Orientierung rasch beurteilt werden. Auch wenn andere Maßnahmen am Patienten vorgenommen werden (z. B. Umlagerung, Monitorisierung, Entkleidung), liefert die Beobachtung des Patienten wichtige Informationen. Bei der Umlagerung oder beim Entkleiden können z. B. allein durch die Beobachtung gewisse neurologische Störungen (z. B. Paresen, Dysdiadochokinese, Ataxie, Tremor, Gangstörung) oder typische Erkrankungsstigmata erkannt werden.

### Untersuchung der Vitalfunktionen: von A bis C

Wann immer ein Patient nicht wach und ansprechbar und noch nicht verlässlich monitorisiert ist, gilt es, die Vitalfunktionen ohne Verzögerung zu überprüfen. Die dabei verwendete Technik ist einfach und als ABC-Schema, abgekürzt für Airway – Atemweg, Breathing – Atmung, Circulation – Kreislauf, bekannt [8]. Die Übergabe eines intubierten Patienten sollte immer mit einem ABC-Check beginnen. Dies ist gerade bei der Übernahme von instabilen Patienten (z. B. Patienten mit Zustand nach kardiopulmonaler Reanimation oder Polytrauma) in der Notaufnahme wichtig, um zu verhindern, dass ein Herz-Kreislauf-Stillstand erst nach der Patientenübergabe erkannt wird und somit wertvolle Zeit und Chancen auf eine gute Prognose verloren werden.

**Figure 5:**
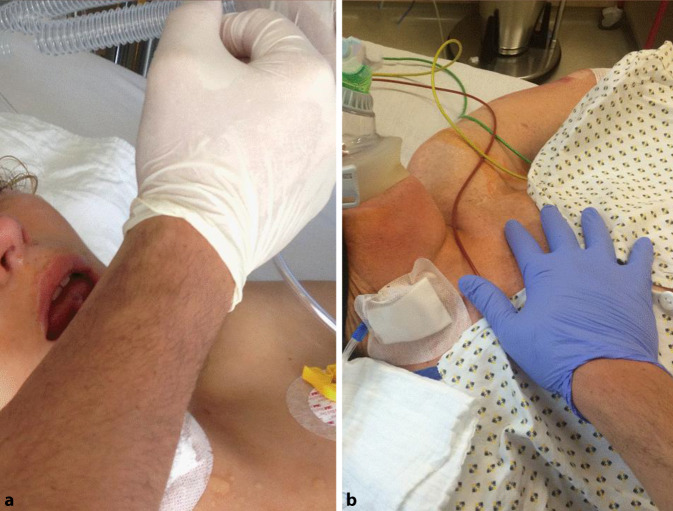

Der Atemweg (A für Airway) wird als erster evaluiert und kann mittels „Sehen“, „Hören“ und „Fühlen“ überprüft werden. Dabei wird das Atemmuster des Patienten visuell inspiziert. Eine paradoxe Atmung, bei der sich der Thorax senkt während sich das Abdomen hebt, weist auf einen verlegten Atemweg hin. Obwohl das Vorhandensein einer physiologischen Atmung, bei der sich der Thorax und das Abdomen gleichzeitig heben und senken, eine Atemwegsverlegung unwahrscheinlich macht, schließt diese eine partielle Atemwegsverlegung nicht sicher aus. Ähnliches gilt für das atemsynchrone Beschlagen einer Sauerstoffmaske. Während ein verlegter Atemweg keine oder nur leise Geräusche verursacht, ist der partiell verlegte Atemweg durch Geräusche wie Gurgeln, Schnarchen oder laute Atemgeräusche zu erkennen. Zuletzt kann die Ausatemluft auch gefühlt werden. In der Praxis hat sich dazu die volare Seite des Unterarms bewährt (Abb. 5a).

Ein verlegter Atemweg verursacht keine oder nur leise Geräusche

Eine Teilverlegung des Atemwegs kann selbst durch diese Techniken nicht ausgeschlossen werden. Zur definitiven Überprüfung empfiehlt sich die Anwendung des HTCL-Manövers („head tilt and chin lift“), durch das der Atemweg komplett geöffnet wird. Nehmen die Atemexkursionen zu und normalisiert sich dadurch das Atemmuster des Patienten, war der Atemweg zuvor partiell verlegt.

Das Vorhandensein der Atmung (B für Breathing) kann nur nach Sicherstellung eines offenen Atemwegs überprüft werden. Hierzu werden wieder Inspektion und Palpation verwendet. Gerade bei oberflächlicher Atmung, die visuell oft schwer bzw. nur an der oberen Thoraxapertur oder durch Bewegungen des Abdomens zu erkennen ist, hat sich die Palpation des Thorax bewährt (Abb. 5b).

Das Vorhandensein eines Kreislaufs (C für Circulation) wird durch die Palpation einer zentralen Arterie (zumeist der Arteria carotis oder Arteria femoralis) überprüft. Um einen zentralen Puls bei Patienten mit Bradykardie nicht zu übersehen, wird empfohlen, die Palpation einer zentralen Arterie für mindestens 5 s, nicht aber länger als 10 s durchzuführen [8].

### Erkennen von präterminalen Zeichen

**Figure 6:**
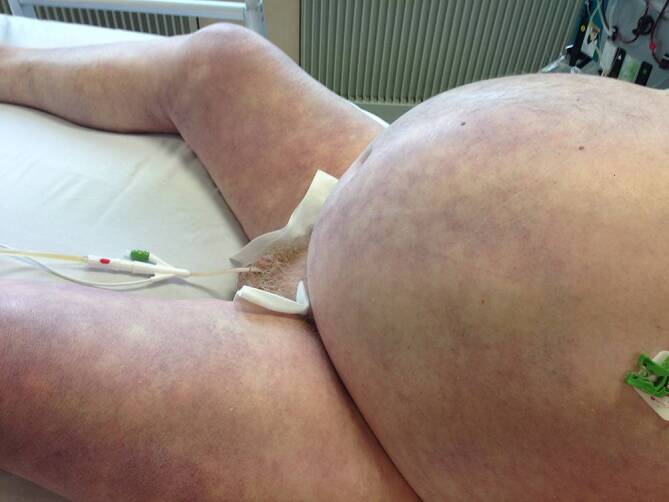

Eine der wichtigsten Prioritäten in der Notfall- und Intensivmedizin ist es, Patienten zu erkennen, die innerhalb kurzer Zeit (Minuten!) einen Herz- und/oder Atemstillstand erleiden werden. In diesen Fällen müssen therapeutische Maßnahmen unmittelbar gesetzt und über alle anderen diagnostischen Schritte priorisiert werden, um dies zu verhindern. Die klinische Untersuchung und das Erkennen von präterminalen Zeichen, also von Symptomen, die häufig einem Atem-Kreislauf-Stillstand vorausgehen (Abb. 6), sind dabei von essenzieller Bedeutung. Tab. 5 fasst die häufigsten dieser präterminalen Zeichen zusammen.

**Table 5:** 

Abfall der Atemfrequenz bei weiterhin bestehenden Zeichen einer respiratorischen Insuffizienz
Bradypnoe <8 Atemzüge/min
Apneustische oder Schnappatmung
Massive Zentralisation (Abb. 6) – unabhängig von gemessenen Blutdruckwerten!
Abfall der Herzfrequenz bei arterieller Hypotonie
Bradykardie <30–35 Schläge/min
Agitation oder Bewusstseinseintrübung bei schwerer respiratorischer oder hämodynamischer Insuffizienz
Parasympathische Symptome (z. B. starkes Schwitzen, Erbrechen, Defäkation, Hypersalivation) bei schwerer respiratorischer oder hämodynamischer Insuffizienz
Cushing-Triade (Bradykardie, arterielle Hypertonie, unregelmäßige Atmung)
Anisokorie
Bilaterale Mydriasis

### Organspezifische Untersuchungsabläufe

Die Abhandlung der detaillierten klinischen Untersuchungsabläufe der wichtigsten Organsysteme (Atemwege und Lunge, Kreislauf, Gehirn, Abdomen, Leber, Nieren, Rückenmark, neuromuskuläres System, Blutgerinnung) in der Notfall- und Intensivmedizin würde den Umfang dieses Beitrags übersteigen. Es wird daher auf die entsprechende Literatur verwiesen [6, 9]. Wichtig ist allerdings zu erwähnen, dass die klinischen Untersuchungsschritte die wichtigsten Funktionen der einzelnen Organsysteme erfassen müssen. Dies kann nur durch einen systematischen und strukturierten Ansatz erreicht werden. Eine unstrukturierte Untersuchungstechnik führt zwangsläufig dazu, dass wichtige Symptome übersehen werden und somit essenzielle therapeutische Interventionen nicht eingeleitet werden können. Nochmals sei an dieser Stelle ausdrücklich erwähnt, dass solche strukturierten klinischen Untersuchungen erlernt und regelmäßig angewandt werden müssen.

## Fazit für die Praxis

- Die Anamnese und klinische Untersuchung stellen das traditionelle Grundwerkzeug der ärztlichen Tätigkeit bei der Diagnosefindung dar.
- Sowohl Anamnese als auch klinische Untersuchung haben in der modernen Notfall- und Intensivmedizin zu Unrecht an praktischem Stellenwert verloren.
- Bei der Diagnosefindung des akut oder kritisch kranken Patienten müssen die Reihenfolge, Technik und Fokussierung der Anamnese und klinischen Untersuchung an die individuelle Situation und den Zustand des Patienten angepasst werden.

## Caption Electronic Supplementary Material


