# Supplementary material for: Medical history taking and clinical examination in emergency and intensive care medicine
Source: Med Klin Intensivmed Notfmed. 2020 Sep 3;115(7):530–8. [Article in German] doi: 10.1007/s00063-020-00731-x (PMC7653795; doi:10.1007/s00063-020-00731-x)
Supplement: Supplementary file 1 [file 63_2020_731_MOESM1_ESM.pdf]

Zusatzmaterial

Leitsymptom-basiertes, strukturiertes Anamneseschema am Beispiel „respiratorische Insuffizienz“

Ja	Nein	Plötzlicher (innerhalb von Sekunden) Beginn der Dyspnoe?
☐	☐	*wenn ja*, erwäge Lungenembolie, Pneumothorax, Anaphylaxie, Inhalation eines Fremdkörpers, rhythmogenes Herzversagen, Trauma

Ja	Nein	Assoziation mit anderen Symptomen?
☐	☐	Brustschmerz<br>*wenn ja*, erwäge myokardiale Ischämie, Lungenembolie, Pneumothorax, Trauma, respiratorische Infektion/ Pleuritis
☐	☐	Palpitationen<br>*wenn ja*, erwäge rhythmogenes Herzversagen
☐	☐	paroxysmale nächtliche Dyspnoe, Nykturie, Gewichtszunahme, bilaterale Beinödeme, Müdigkeit<br>*wenn ja*, erwäge akut-auf-chronisches Herzversagen
☐	☐	Bein-/ Knöchelschwellung<br>*wenn einseitig*, erwäge Lungenembolie; *wenn beidseitig*, erwäge akut-auf-chronisches Herzversagen, Flüssigkeitsüberladung/ Hypervolämie
☐	☐	obstruktive Atmung<br>*wenn ja*, erwäge Asthma/ COPD, Herzversagen, Flüssigkeitsüberladung/ Hypervolämie
☐	☐	Fieber, Schüttelfrost<br>*wenn ja*, erwäge (respiratorische) Infektion und/ oder Sepsis
☐	☐	produktiver Husten, missfarbenes Sputum<br>*wenn ja*, erwäge respiratorische Infektion
☐	☐	Fieber, Gewichtsverlust, Nachtschweiß<br>*wenn ja*, erwäge Tuberkulose, respiratorische Infektion infolge einer hämatologischen Malignität
☐	☐	Hämoptyse<br>*wenn ja*, erwäge Blutung aus Lungentumor, Gefäßmalformation, respiratorische Infektion (Tuberkulose, Pneumokokkenpneumonie). Bronchiektase, Lungenembolie, diffuse alveoläre Blutung

Ja	Nein	**Symptomerleichterung durch Änderung der Körperposition?**
☐	☐	Orthopnoe<br>*wenn ja*, erwäge Herzversagen, Flüssigkeitsüberladung/ Hypervolämie, respiratorische Infektion
☐	☐	sitzende Position mit (auf die Knie) aufgestützten Armen<br>*wenn ja*, erwäge COPD/ Asthma
☐	☐	Trepopnoe<br>*wenn ja*, erwäge Herzversagen, einseitige Lungenerkrankung (z.B. Pleuraerguss)
☐	☐	Platypnoe<br>*wenn ja*, erwäge basale Pneumonie oder Lungenemphysem, hepatopulmonales Syndrom

Ja	Nein	**Vorerkrankungen?**
☐	☐	chronisches Lungen-, Herz- oder Niereninsuffizienz<br>*wenn ja*, erwäge Exazerbation der Grunderkrankung
☐	☐	Malignom<br>*wenn ja*, erwäge Pleuraerguss, respiratorische Infektion, Lungenembolie, Lungenmetastasen, Strahlen-induzierter Lungenschaden
☐	☐	Allergie/ Atopie<br>*wenn ja*, erwäge Asthma, Anaphylaxie
☐	☐	Z.n. Beinvenenthrombose oder Lungenembolie<br>*wenn ja*, erwäge Lungenembolie
☐	☐	rezente respiratorische Infektion<br>*wenn ja*, erwäge protrahierte oder neue respiratorische Infektion
☐	☐	rezentes Thoraxtrauma<br>*wenn ja*, erwäge muskuloskelettale Verletzung, Lungenkontusion, Pneumo- oder Hämatothorax
☐	☐	HIV Infektion<br>*wenn ja*, erwäge respiratorische Infektion (inkl. Tuberkulose und Pneumozystis Infektion) und/ oder Pneumothorax (insbesondere bei Pneumozystis Infektion)

Ja	Nein	**Medikamentenanamnese?**
□	□	kurz- oder längerfristige Einnahme von einem oder mehrerer der folgenden Medikamente: Amiodaron, Bleomycin, Mitomycin C, all-trans Retinoinsäure, Cytarabin, Amphotericin, Carbamazepin, Azathioprin, Methotrexat, Phenytoin, Busulfan, Taxane, Gold, Taxan-beinhaltende Medikamente/ Implantate (inkl. Stents)<br>*wenn ja*, erwäge Medikamenten- induzierte Lungenschädigung

Ja	Nein	**Berufliche Tätigkeit?**
□	□	Staubexposition (z.B. Asbest, Kohle, Quarzstaub, etc.)<br>*wenn ja,* erwäge sekundäre pulmonale Fibrose oder respiratorische Infektion (inkl. Tuberkulose bei Quarzstaubexposition)

Ja	Nein	**Sozialanamnese?**
□	□	Raucher<br>*wenn ja*, erwäge COPD, respiratorische Infektion, Herzinsuffizienz, Lungenkarzinom oder Pneumothorax
□	□	übermäßiger Alkoholkonsum<br>*wenn ja*, erwäge respiratorische Infektion oder Aspirationspneumonie

Ja	Nein	**Familienanamnese**
□	□	Allergie/ Atopie<br>*wenn ja*, erwäge Asthma
□	□	Asthma<br>*wenn ja*, erwäge Asthma
□	□	gentisch-bedingte Lungenerkrankungen (z.B. zystische Fibrose, Alpha1-Antitrypsin-Mangel)<br>*wenn ja*, erwäge genetisch bedingte Lungenerkrankung
□	□	Tuberkuloseinfektion<br>*wenn ja,* erwäge Tuberkulose
